# Citizen science in data and resource-limited areas: A tool to detect long-term ecosystem changes

**DOI:** 10.1371/journal.pone.0210007

**Published:** 2019-01-09

**Authors:** Adam Gouraguine, Joan Moranta, Ana Ruiz-Frau, Hilmar Hinz, Olga Reñones, Sebastian C. A. Ferse, Jamaluddin Jompa, David J. Smith

**Affiliations:** 1 University of Essex, School of Biological Sciences, Colchester, United Kingdom; 2 Instituto Español de Oceanografía (IEO), Centre Oceanogràfic de les Balears, Ecosystem Oceanography Group (GRECO), Moll de Ponent sn, Palma, Spain; 3 Department of Ecology and Marine Resources, Instituto Mediterráneo de Estudios Avanzados IMEDEA (CSIC-UIB), Esporles, Spain; 4 Ecology Department, Leibniz Centre for Tropical Marine Research (ZMT), Bremen, Germany; 5 Faculty of Biology & Chemistry (FB2), University of Bremen, Bremen, Germany; 6 Center for Coral Reef Research, Hasanuddin University, Makassar, Indonesia; Tanzania Fisheries Research Institute, UNITED REPUBLIC OF TANZANIA

## Abstract

Coral reefs are threatened by numerous global and local stressors. In the face of predicted large-scale coral degradation over the coming decades, the importance of long-term monitoring of stress-induced ecosystem changes has been widely recognised. In areas where sustained funding is unavailable, citizen science monitoring has the potential to be a powerful alternative to conventional monitoring programmes. In this study we used data collected by volunteers in Southeast Sulawesi (Indonesia), to demonstrate the potential of marine citizen science programmes to provide scientifically sound information necessary for detecting ecosystem changes in areas where no alternative data are available. Data were collected annually between 2002 and 2012 and consisted of percent benthic biotic and abiotic cover and fish counts. Analyses revealed long-term coral reef ecosystem change. We observed a continuous decline of hard coral, which in turn had a significant effect on the associated fishes, at community, family and species levels. We provide evidence of the importance of marine citizen science programmes in detecting long-term ecosystem change as an effective way of delivering conservation data to local government and national agencies. This is particularly true for areas where funding for monitoring is unavailable, resulting in an absence of ecological data. For citizen science data to contribute to ecological monitoring and local decision-making, the data collection protocols need to adhere to sound scientific standards, and protocols for data evaluation need to be available to local stakeholders. Here, we describe the monitoring design, data treatment and statistical analyses to be used as potential guidelines in future marine citizen science projects.

## Introduction

In the face of predicted large-scale coral degradation over the coming decades [1], it is crucial to establish long-term data series to explore how the degradation of corals can affect associated ecosystem components and the implications of those changes for human well-being [2]. Coral reefs are threatened by a range of impacts, resulting in coral loss at global and local scales [3]. Increasing sea surface temperature (SST) has been identified as the principal threat to corals globally, causing thermally induced coral beaching often resulting in coral mortality and subsequent breakdown of the reef matrix [4]. At a local scale, coral reef degradation is mainly the result of growing coastal populations and a range of associated stressors. Impacts such as the overharvesting of reef fish, the use of destructive fishing techniques, sewage, industrial pollution, sedimentation and recreational SCUBA diving can inflict severe damage to coral over short periods of time [3]. Subsequently, the degradation of coral following disturbance events can alter the composition of the associated fish communities by affecting species reliant on the reef structural matrix for shelter and those requiring live corals as a food resource [5]. The loss of essential habitat often has a significant impact on the abundance of small, coral-dwelling fish (e.g. Pomacentridae and Pseudochromidae), which due to their size rely on the coral structural matrix for shelter. Reduction in food availability caused by the loss of live coral most commonly affects coral-feeding fish such as Chaetodontidae and Pomacanthidae [6,7].

The need for long-term data on ecosystem changes has long been recognised, not only for coral reefs, but also for other ecosystems, highlighting the importance of the few existing datasets [8]. The establishment and maintenance of environmental monitoring programmes, however, requires long-term commitment of funding institutions and/or governments at national or regional levels. While sustained funding might be easier to achieve in economically and politically stable countries, it is not always guaranteed. In times of financial crises, cuts in government spending in developed countries commonly target environmental conservation schemes, often reducing and curbing established monitoring programmes [9]. On the other hand, in areas where economic resources are limited, political scenarios might frequently change and where environmental monitoring is not perceived as a priority, long-term monitoring can simply be absent altogether. Thus, when and where sustained investment might not be an option, the use of citizen science emerges as a powerful alternative for setup and maintenance of long-term monitoring programmes. Citizen science, i.e. the use of volunteers or public participation in scientific research, has been acknowledged as a useful addition to both conservation and ecology as it can generate data at broad spatial and temporal scales [8,10].

Since ecosystem vulnerability is generally context-dependent, as is the case for coral reefs [3], it is also important to expand assessment and monitoring techniques so that a broader range of systems across gradients of anthropogenic and natural multi-stressors are examined. Only then will we be able to construct realistic predictions of how these ecosystems might react to future disturbances. Furthermore, while some coral reef habitats have been well characterised [11], many have not [12]. This lack of information for some areas of the world represents a challenge for the development of ecological predictions as there is evidence that coral reefs, depending e.g. on their spatial isolation [13], can significantly differ between areas, much more than what natural processes would predict [14]. This implies that reef communities, biodiversity and therefore species interaction networks can be significantly different between areas. It is therefore important to find alternative approaches to enable the acquisition of ecological knowledge to fill these gaps.

In this paper we want to draw attention towards the potential of citizen science programmes in detecting ecosystem change in areas where sustained research funding is lacking and highlight its importance in enlarging the pool of ecological data to areas likely to remain unexplored through traditional research. Data collected through citizen science programmes can play a crucial part in the generation of comprehensive global datasets, providing essential information where otherwise no data would be available. Not all citizen science projects, however, have been able to contribute to the long-term monitoring of ecosystems; in fact only a small proportion of citizen science data ultimately make it to peer-reviewed scientific articles [15]. Poor data quality and failure to analyse it using sound statistical methods are some of the frequent citizen science data challenges [16,17]. For citizen science data to contribute to ecological monitoring in a meaningful way, the data collection protocols need to adhere to sound scientific standards and need to be available to stakeholders interested in continuing or extending the collection or analysis of data. Thus, in addition, we describe the monitoring design, data treatment and statistical analyses to be used as potential guidelines in future marine citizen science projects.

We used an 11-year coral reef monitoring dataset collected by volunteers in a previously poorly explored area in Southeast Sulawesi (Indonesia) to emphasise that citizen science programmes are an essential resource in biodiversity research and demonstrate how they can generate much needed data for the detection of long-term ecosystem change in areas where no other information might exist.

## Materials and methods

### Study site

**Fig 1:**
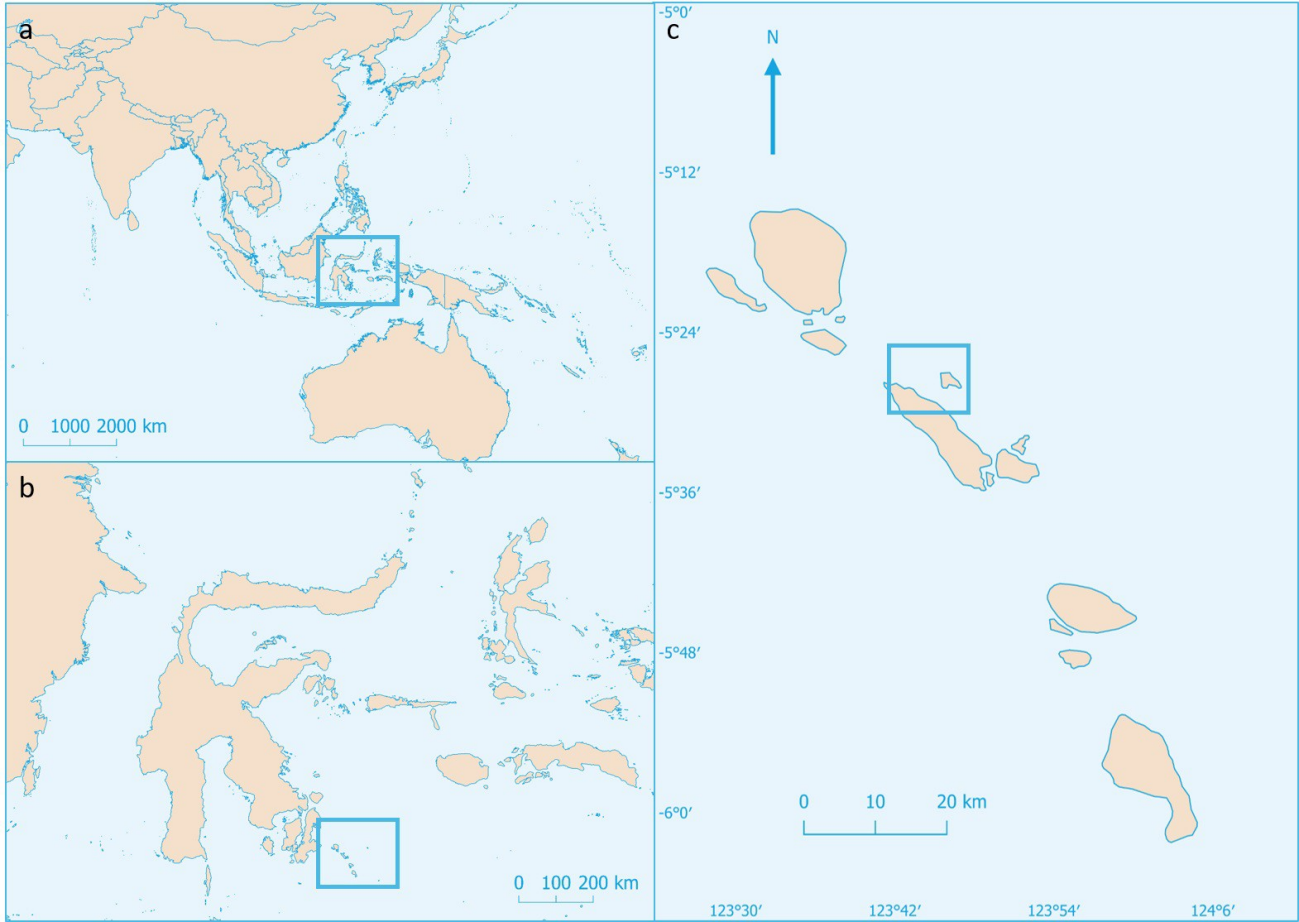
Sampling location. Boxed areas represent: a) Sulawesi, b) Wakatobi National Park, c) monitoring area.

Sampling took place on some of the richest and most diverse coral reefs in the world, located around the islands of Hoga and Kaledupa, within Wakatobi National Park (WNP), in the Tukang Besi archipelago in South East Sulawesi, Indonesia (Fig 1 and Table 1) [12]. With a surface area of 13,900km^2^ and a resident community of around 100,000 people, WNP is the one of the largest and the most populated national park in Indonesia [18]. It has historically been characterized by a lack of sufficient funding, ineffective enforcement, minimal community participation in management activities and inappropriate zonation. Local people of WNP are highly dependent upon marine resources, resulting in widespread overfishing and unsustainable exploitation [18]. It has been reported in the literature that bomb fishing is an important fishing practice within the WNP [18], personal observations of which were also made by several of the authors at most of the study sites during the monitoring period.

**Table 1:** Names and geographic coordinates of the sites sampled.

	GPS coordinates	
Site	South	East
Pak Kasim	05°27'.925''	123°45.323''
Ridge	05°26'. 940'	123°45'. 245'
KDS	05°27'.849''	123°42.231''
Kaledupa	05°28'.272''	123°43.429''
Buoy 3	05°28'.380''	123°45.451''
Sampela	05°29'.062''	123°45.228''

### Long-term monitoring

A long-term monitoring programme was implemented in WNP in 2002 through collaboration between Operation Wallacea and the Indonesian Institute of Sciences (LIPI) [18]. Operation Wallacea is a network of foreign and local Indonesian academics, who design and implement biodiversity and conservation management research expeditions supported by volunteers. Volunteers, mainly university students, bear all the costs of their participation in the programme, including travel, accommodation and associated training expenses. The average number of volunteers involved with the WNP monitoring programme for any given year is approximately 25 and the typical length of their stay is four weeks. Different aspects of the programme (e.g. education, training, logistics) are run by the visiting academic staff, as well as members of the local community. Data collected are subsequently shared with the local government, the National Parks Agency, the Indonesian Institute of Sciences and the Indonesian Ministry of Research, Technology and Higher Education.

### Data collection

The temporal variation in benthic cover and the associated fish community structure was assessed by a mix of academic staff and volunteers, yearly from June to August, between 2002 and 2012, by surveying 51 permanent transects of 50m length. The transects were established in the study area using a nested design, in replicates of three (except at one location where there was no reef flat habitat), on the reef flat (5m horizontal distance on the landward side from the reef crest), the reef crest and the upper reef slope (defined by habitat type and a depth of 10m). At the start and the end, permanent transects were marked with steel pickets, and each transect was separated by a minimum horizontal distance of 20m. Using Continuous Line Intercept Transect technique, a single diver recorded the % cover of different benthic biotic (hard and soft coral, macro algae) and abiotic (rock, rubble, sand) categories, while fish counts were carried out by single-diver underwater visual censuses [19]. The same transects were used for benthic and fish surveys. The sampling was conducted between the hours of 09.00 and 16.00, excluding the high activity periods of early morning and late afternoon, thus reducing variability in fish densities due to diurnal influence on behaviour [20]. A total of 495 transects were sampled over 11 years. Due to logistical constraints not all permanent transects established were sampled each year. In years 2004, 2006 and 2007 transect samples missed represented 49%, 33% and 22% of the total possible combinations of year by locations, respectively. For the remaining years in which transects were missed (2002, 2003, 2008 and 2009), they represented less than 10% of the total possible combinations. Overall, almost an identical number of transects was sampled for crest and slope habitats, while the reef flat habitat was represented by 27% less transects.

To investigate if any of the trends observed in benthic cover were related to high temperature anomalies, we obtained area-specific monthly means Extended Reconstructed Sea Surface Temperatures (ERSST) from the National Oceanic and Atmospheric Administration (NOAA) website (https://www.nesdis.noaa.gov/). To obtain information about the coral bleaching temperature threshold in the sampling area we used the Regional Virtual Station for South East Sulawesi [21]. Thermal stress likely to cause significant coral bleaching was determined by identifying the months, termed Degree Heating Months (DHM), in which the monthly temperature mean exceeded the coral bleaching temperature threshold [22].

### Volunteer training and data treatment

Between 2002 and 2012, approximately 275 volunteers, supervised by experienced researchers, collected the study data. Volunteers did not necessarily have previous diving and/or sampling experience. If the volunteers were not SCUBA proficient, they completed a PADI Open Water course at the local dive centre. Subsequently, they attended a one-week reef ecology course specific to the sampling area, which combined theory and field sessions on the identification of different benthic categories and fish species. To participate in the data collection, volunteers had to achieve a minimum of 90% in a test at the end of their training period. To improve data quality, volunteers’ first identifications were cross-checked against those of experienced researchers.

To reduce observer-related variability we introduced various data treatment procedures minimising the presence of any erroneous or false data. All the fish species which occurred in a single transect and did not reappear in previous or subsequent years were removed. By doing so, there was a risk of removing some of the rare species from the dataset. However, due to the extremely large number of species and relatively low experience of the observers, it was more likely that these species were indeed erroneously identified and including them would have introduced large variability in the dataset. We used FishBase (http://www.fishbase.org/) to match the species’ geographical distributions–if a species recorded had previously not been observed in our study area it was marked as a possible erroneous identification. All possibly erroneous species were photographically compared to the species within the dataset with confirmed occurrences in the sampling area, and their abundances were added to the physically most similar species of the same fish family. Finally, because the initial programme design did not include cryptic and transient pelagic species, those accidentally recorded by the volunteers were also removed.

### Statistical analyses

To observe temporal sequential change in fish community composition, nonmetric multidimensional scaling (MDS) was performed on annual mean abundance data per year, using software PRIMER v6 [23]. The resulting ordination was tested for seriation to identify a statistically significant sequential pattern with consecutive years having a higher similarity compared to years separated by longer time periods. The Simprof test was applied to ascertain if some years formed statistically significant clusters (year groups) due to their similarity in species composition.

In line with recommended statistical solutions for error and bias in global citizen science datasets, we used Poisson or negative binomial generalized linear mixed-effects models (GLMMs) to test the degree of influence of hard coral cover on the fish community abundance and species richness [16]. Zero-inflated Poisson or Zero-inflated negative binomial generalized linear mixed models (Zero-inflated GLMMs) were used to investigate the relationship of hard coral cover and fish family abundance [16]. If the relationship was statistically significant and if the fish family abundance contributed to >1% of the total community abundance, individual species within the family were also tested for their hard coral cover–abundance relationship. Due to the large number of species within families, we tested the relationships for all species which cumulatively attributed to ≥ 90% of the total family abundance contribution. We thus excluded occasional, possibly erroneously identified and non-dominant species, and included statistically significant hard coral cover–abundance relationships of all other species. We chose Zero-inflated GLMMs for these analyses as zero inflation was evident in the transect data. All models were random intercept models where the random factor was Year. We analysed all model data with the package glmmTMB in the statistical software R [24,25].

## Results

### Data quality

Benthic data collected was of consistent quality, as the surveys were based on easily distinguishable broad benthic categories. Conversely, fish data showed a much higher variability. This was mainly due to two main factors: the large number of fish species and difficulty to correctly identify and count them, and the collection of data by a multitude of observers of different experience levels.

Removal of the fish species which occurred in a single transect and did not reappear in previous or subsequent years amounted to approximately 35% of all species but had less than 1% contribution to the total abundance. By conducting photographic comparisons of potentially erroneously identified species to the species with confirmed occurrences in the sampling area we further reduced the species variability by 20% from the initial dataset, however we preserved the overall abundance. By identifying erroneously recorded cryptic and transient pelagic species we eliminated 4% of the fish species and reduced the total fish abundance by < 0.1%.

### Benthic community and temperature anomalies

**Fig 2:**
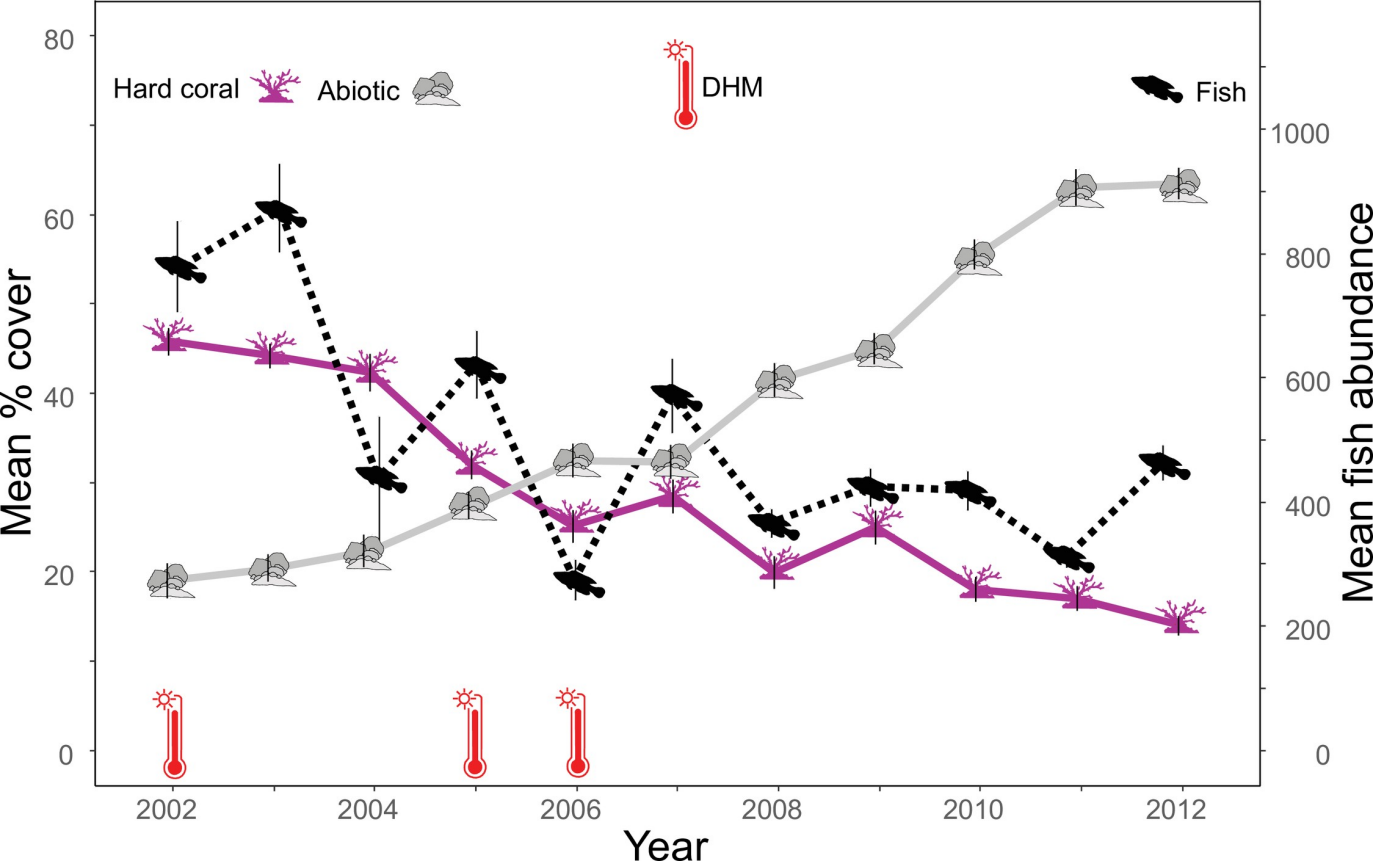
Yearly changes per transect in mean fish community abundance and mean cover of hard coral and abiotic benthos (vertical lines represent standard error). Occurrence of one Degree Heating Month (DHM) displayed for each corresponding year.

The reefs in 2002 were characterised by high hard coral cover and low abiotic cover (Fig 2). Subsequently, annual surveys documented a continuous decline from the initial mean hard coral cover of 45.8% in 2002 to a low mean of 14% in 2012. We observed the most notable changes between 2004 and 2005 with 10.3% decline, and between 2007 and 2008, when mean hard coral cover declined by 8.5%. Overall, we evidenced a decline of 69.3% in mean hard coral over the 11-year period, relative to the initial cover. Conversely, over the same period the mean abiotic cover increased from 19% in 2002 to 63.4% in 2012. Algae experienced an increase in mean cover from 16.4% in 2002 to 22.5% in 2006, followed by a gradual decrease to 4.9% in 2012 (S1 Fig). Mean cover of soft coral demonstrated little fluctuation and appeared to be relatively stable over the study period; 15.6% in 2002 and 13.6% in 2012 (S1 Fig).

According to the South East Sulawesi Coral Reef Watch Regional Virtual Station the bleaching temperature threshold for the area is 30.38°C. Consequently, using the ERSST data, we identified three years with DHM (2002, 2005 and 2006) (Fig 2).

### Fish community

**Fig 3:**
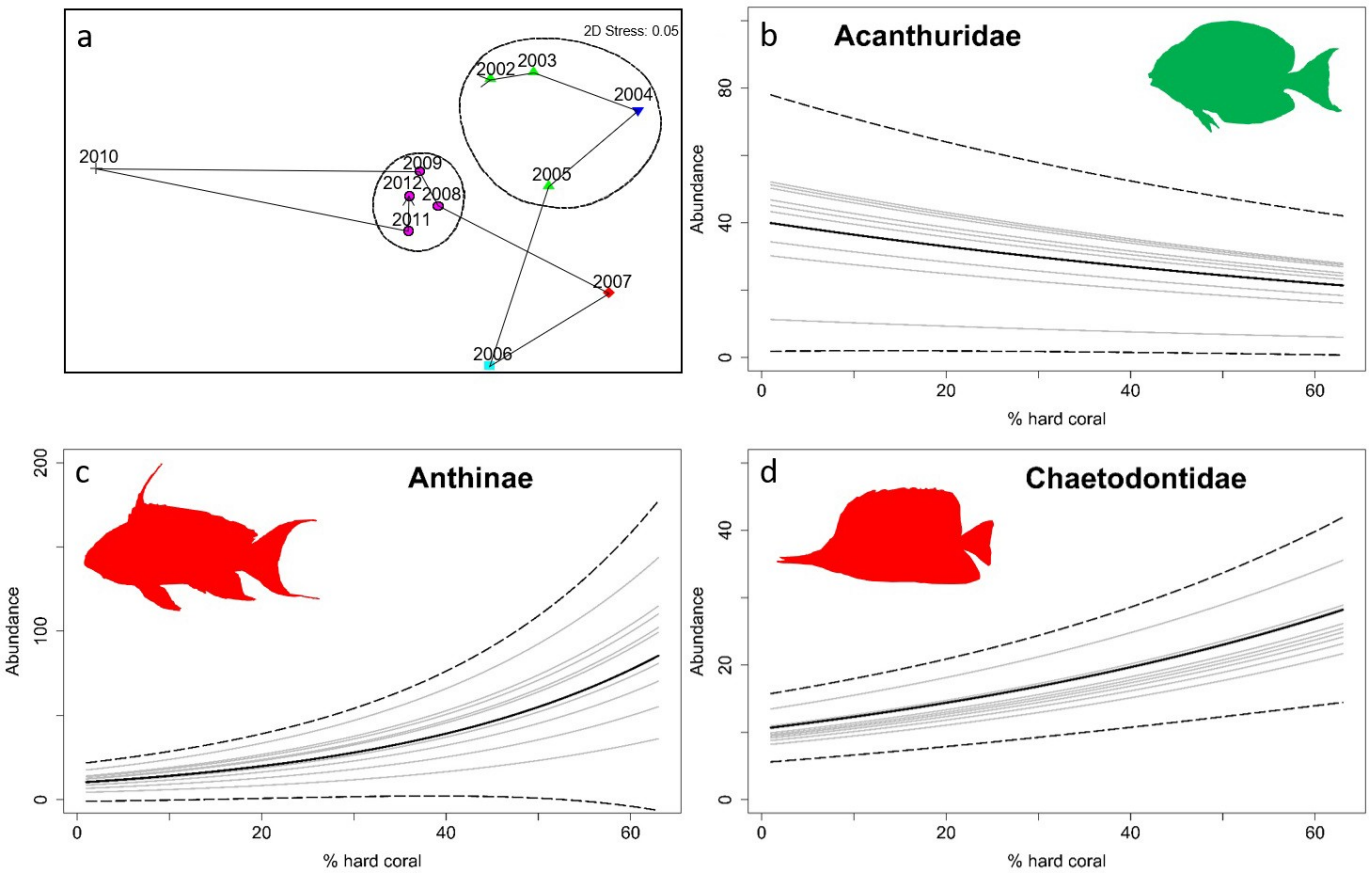
**a)** MDS ordination plot with years sequentially connected by an arrow. Different colours and shapes represent years classified by the Simprof routine as statistically significant year groups. The circles represent a similarity level of 50%; **b, c & d)** Zero-inflated negative binomial generalized linear mixed effect models fitted to the relationship between abundance and hard coral cover for each year separately (solid grey lines) and mean of all years (solid black line), and confidence intervals of the mean (dashed black lines) for Acanthuridae, Anthiinae, Chaetodontidae, respectively. (Graphical representations of b, c & d families based on particularly strong and ecologically significant relationships with hard coral cover, those of all other families available in S3 and S4 Figs).

The seriation test on the nonmetric MDS representing the evolution of fish community composition through time showed that a significant sequential pattern existed in the fish community composition (Rho = 0.24, p = 0.03). The Simprof test identified significant year groupings at p<0.05 (Fig 3A). The 1^st^ grouping comprised the years 2002, 2003 and 2005, and the 2^nd^ one the years 2008, 2009, 2011 and 2012. Year 2004 did not belong to the groups identified by Simprof, but clustered more closely to the 1^st^ group. The two-year groupings were connected by years which were notably distinct from both groups, 2006 and 2007, representing a transition period, while 2010 was highly distinct from all other years. The two groupings and the transition years match the temporal trend in hard coral cover, coinciding with initial years of high hard coral cover (1^st^ grouping), followed by transition years and culminating in years of low hard coral cover (2^nd^ grouping).

We observed significant negative effects of decreasing hard coral cover on the fish community abundance and species richness (S2 Fig). Furthermore, we identified significant abundance relationships with hard coral cover for 13 fish families. Acanthuridae (Fig 3B), Nemipteridae, Pomacanthidae and Epinephelinae displayed a negative effect of increasing hard coral cover on abundance. Hard coral cover had a positive effect on the abundance of Anthiinae (Fig 3C), Balistidae, Chaetodontidae (Fig 3D), Holocentridae, Labridae, Lutjanidae, Pomacentridae, Pseudochromidae and Zanclidae (S1 Table and S3 and S4 Figs). Of the 13 fish families, eight had >1% fish community abundance contribution and subsequently within each, several species with significant hard coral cover–abundance relationship were identified (S2 Table and S5 Fig). The most notable relationships included a highly negative abundance relationship with increasing coral cover for *Ctenochaetus striatus* (Acanthuridae), and highly positive relationships for *Pseudoanthias squamipinnis* and *Forcipiger flavissimus*, representative of the Anthiinae and Chaetodonidae, respectively.

## Discussion

This study provides evidence of the importance of citizen science programmes in detecting long-term ecosystem change. Using the volunteer collected data, we observed a profound alteration in benthic habitat, as well as its effect on the associated fish fauna. At the community level, fish abundance decreased in parallel to decreasing hard coral cover, resulting in a fish community much different in abundance and species composition at the end of the monitoring period compared to the beginning. While the loss of hard coral cover had a strong effect on fish community abundance, the effect on community richness was subtle. Nonetheless, with a decrease in hard coral cover the number of species also decreased. In addition to reduction in hard coral cover, it is likely that bomb fishing and an increasing human population, dependent on fish for food, were also contributing factors driving fish community changes.

At the fish family level, the data showed that not all fishes responded in the same way, and both positive and negative relationships with hard coral cover were detected. Anthiinae (a subfamily of the Serranidae) showed a strong negative relationship with decreasing coral cover. Most species of this subfamily live close to the substratum and due to their small size rely on the coral structural matrix for shelter [5]. The decline in their abundances could have been attributed to the loss of habitat caused by bomb fishing, resulting in direct structural collapse of the reef matrix. Other families of small-bodied fishes observed in this study also experienced decreases in abundance (e.g. Pomacentridae, Pseudochromidae). Prior to 2006, the most dominant species were exclusively made up of members of small-bodied, coral-reliant fish families. After 2006, loss of dominance of certain small-bodied families and their replacement by the species of other families was observed. This further supports the argument of the reliance of these families on coral habitat since the shift in species dominance occurred at a point at which the benthic habitat experienced a profound hard coral cover loss. Besides a loss of essential habitat for small-bodied coral-dwellers, reduction in hard coral can also cause reduction in food availability for coral-feeding fishes [5]. Here, these fishes were represented by the Chaetodontidae, displaying a strong negative relationship with decreasing coral cover. Concordant with these findings, there is a large body of evidence also confirming the positive correlation between the abundance of Chaetodontidae and the amount of hard coral available [6,7]. There is some evidence, however, that hard coral cover is not always the most important limiting factor for the abundance of Chaetodontidae and the effects could be species-dependent [26].

Certain fish families, however, increased in abundances following decreases in coral cover. Acanthuridae were the most prominent example. Most Acanthuridae are detritivorous, feeding on loose sediment [27]. Reduction in coral cover was accompanied by an increase in abiotic cover, thereby increasing the food availability for detritivorous fish. Furthermore, the abundance contribution of the species pointed to a shift in dominance over years, with the appearance of Acanthuridae in 2006 and a decrease in dominance of previously higher ranked Pomacentridae. The period of change in the fish community structure corresponded to that of large decreases in hard coral cover and increases in abiotic cover. Increase in Acanthuridae following reduction in hard coral cover has also been reported in previous studies (e.g. [28]), however there also are those which failed to detect such a relationship [29,30]. Pomacanthidae and Nemipteridae also experienced increases in numbers. Species belonging to these families commonly feed on highly mobile invertebrates and small benthic fish and hence are not strongly dependent on live coral for food or habitat [31]. Furthermore, they may have also benefited from increases in abiotic cover, primarily rubble, housing many larval fish and invertebrates.

Although there are no reports in the literature of any mass bleaching events for the study area, the ERSST data confirmed occurrence of DHMs in 2002, 2005 and 2006, which likely contributed to the reduction in hard coral cover observed. The reduction in coral might have also been driven by other important factors. Bomb fishing, causing physical destruction of coral, was identified as one of the most prevalent fishing practices used within the WNP [18]. Since the population living in the already densely inhabited study area increased over the monitoring period, coupled with the high dependency on reef fish for food, this could have induced considerable physical damage to the reef from destructive fishing practices [18]. Personal observations of the blast fishing damage to the coral, as well as hearings of explosions in the proximity, were made throughout the monitoring period. Finally, fast coral disease progression rates and high tissue mortality rates for coral diseases have also been reported on many of the sites sampled in the park [32].

Citizen science projects provide conservation, ecological science and management an extremely powerful tool that allows the collection of scientifically reliable data at temporal and spatial scales, which would be otherwise unfeasible [33]. So far however, citizen science projects have often focused on terrestrial ecosystems within developed nations. Of the 509 citizen science initiatives described by Pocock et al. in their systematic review, only 75 projects were marine and of those, all but one took place in Europe or the United States [34].

With this study, we wanted to draw attention towards the unexplored potential that citizen science has for developing nations, where the establishment and maintenance of long-term data collection programmes might not be feasible and government agencies are often poorly equipped or trained to carry out necessary monitoring. Data collected through citizen science can subsequently, as is the case here, be transferred to local government and national agencies (e.g. science, conservation and education agencies) to inform evidence-based environmental management and conservation strategies. During the initial stages of the programme, citizen science programmes rely on sustained involvement of scientists to ensure consistent quality of the data collected. However, once the programme is set-up and the periodic data collected deemed sufficiently robust and meaningful, scientists should build easy-to-follow data management protocols and statistical analysis codes for open access software. These can then be left for the volunteer organization and/or locals to use in the future, cutting the total costs of the programme, aiding in local independency from external sources and knowledge colonialism. The involvement of locals is of particular importance since it has been shown that citizen science can promote scientific literacy and encourage marine stewardship [35]. In return, this can have positive consequences for the environment through changes in behaviour and an increase in environmental awareness [36]. Arguably in the present case study, a large proportion of the participants originated from developed nations, having the financial capacity to pay for their trip and subsistence costs during the stay. As such, the project could be viewed as citizen science through “volunteer tourism” [37]. We argue that volunteer tourism is a form of citizen science in the sense that data collection is carried out by non-experts on a volunteer basis, therefore fitting under the citizen science umbrella term, and additionally containing some particularities that might not be shared through all citizen science projects. Generally, volunteer tourists incur non-negligible costs to participate in research or conservation projects, which normally take place in exotic locations, targeting charismatic ecosystems. These aspects contrast with the nominal costs that might be incurred by the majority of citizen science volunteers and the opportunity to collect data in “their own backyards” [38]. The differentiating elements, however, do not exclude these projects from providing data on global environmental problems, therefore contributing to the social and political discourse required to tackle global challenges. As such, volunteers participating in volunteer tourism projects could be viewed as citizen scientists who hold feelings of global citizenship and global environmental stewardship, facilitating research on a local scale for problems that might not necessarily affect them [38]. However, one must be aware that there also negative aspects associated to volunteer tourism and that the drive behind volunteers is not always related to environmental reasons, as it has been noted in a number of volunteer tourism projects that volunteers were primarily interested in personal gain, rather than conservation [39]. Furthermore, volunteer tourism can sometimes be profit-tailored by the private business organising the project, often neglecting the needs of the communities and in some circumstances even creating a dependency on this type of tourism [37,40]. Volunteer tourism is thus not flawless, requiring therefore a critical analysis of the possible negative impacts associated [41]. Additionally, when developing citizen science projects that are primarily based on financially privileged non-locals, projects should take care in avoiding knowledge colonialism, whereby the knowledge is created solely by the wealthy and provided as a gift to the less well-off [40]. Integrating such projects into the local communities therefore has to be a key aspect [38].

Frequently, the use of citizen science has been criticised based on the quality of the data collected by non-professionals. In this monitoring programme, a high degree of care was taken to eliminate potential bias associated with inexperienced taxonomists by carrying out training and testing prior to the surveys, collection of data appropriate for the skill levels of the volunteers, as well as subsequent data cross-checks with experienced researchers. These aspects have been shown to improve data quality and ultimately produce data suitable for scientific analysis [42,43]. Additionally, a scientific experimental design was adopted for data collection, ensuring the representativeness of the data. This aspect is not always reflected in citizen science projects, where volunteers are often asked to record the presence or number of particular species in particular areas in an opportunistic manner, without necessarily following a statistically sound design [44,45]. Even so, it is probable that misidentification of certain species occurred, but as we demonstrated, there are multiple ways of treating and subsequently analysing such data to ensure that the ecological interpretation of results is as robust as possible, and that the quality of the data is known and accounted for. If the trend of interest is strong, data variability encountered in citizen science datasets can often be effectively overcome, because of the large quantity of data provided, in contrast to the sometimes-limited quantity of data from more formal surveys. Arguably then, data collected by citizen scientists is not a panacea but a cost-effective way of delivering the base information needed to inform better management.

Within our study, we demonstrate that volunteer monitoring projects can provide useful data and be a powerful tool to gain data for areas that have limited financial governmental support. The limitations of such volunteer tourism approaches, besides the data quality issues discussed, is that they are likely to work only in areas with adequate access, living conditions, and social as well as political stability. Furthermore, these remote areas need to be characterised by sufficiently charismatic ecosystems to persuade volunteers to invest in their trip and participate in such projects. It is improbable that sufficient numbers of altruistic global citizens exist who would travel to unattractive or degraded locations to sustain long-term observations. On the upside, many of the world’s biodiversity hotspots, such as coral reefs, are also environmentally attractive, and as such global citizen science or volunteer tourism projects have an important part to play in the management and conservation of our global environmental heritage, especially in resource limited areas.

## Supporting information

S1 Fig Yearly changes per transect in mean fish community abundance and mean cover of hard coral, algae, soft coral and abiotic benthos (vertical lines represent standard error). Occurrence of one Degree Heating Month (DHM) displayed for each corresponding year.
(DOCX)

S2 Fig Negative binomial generalized linear mixed effect model fitted to the relationship between fish community a) abundance and b) species richness and hard coral cover for each year separately (solid grey lines) and mean of all years (solid black line) and confidence intervals of the mean (dashed black lines). Grey circles represent data points.
(DOCX)

S3 Fig **Zero-inflated negative binomial generalized linear mixed effect models fitted to the relationship between fish families’ abundance and hard coral cover for each year separately (solid grey lines) and mean of all years (solid black line) and confidence intervals of the mean (dashed black lines):** A) Acanthuridae, B) Anthiinae, C) Balistidae, D) Chaetodontidae, E) Epinephelinae, F) Holocentridae, G) Labridae, H) Lutjanidae.
(DOCX)

S4 Fig **Zero-inflated negative binomial generalized linear mixed effect models fitted to the relationship between fish families’ abundance and hard coral cover for each year separately (solid grey lines) and mean of all years (solid black line) and confidence intervals of the mean (dashed black lines):** A) Nemipteridae, B) Pomacanthidae, C) Pomacentridae, D) Pseudochromidae, E) Zanclidae.
(DOCX)

S5 Fig **Statistically significant zero-inflated negative binomial generalized linear mixed effect models for the relationship between species’ abundance and hard coral cover for each year separately (solid grey lines) and mean of all years (solid black line) with the mean confidence intervals (dashed black lines):** A) Acanthuridae, B) Anthinae, C) Balistidae, D) Chaetodontidae, E) Labridae, F) Pomacanthidae, G) Pomacentridae and H) Pseudochromidae species.
(DOCX)

S1 Table Summary of zero-inflated negative binomial generalized linear mixed effect model showing the effect of hard coral cover and year on fish family abundance. The table shows the best-selected model indicating parameter means with standard errors for fixed effects mean (FEM), and variance terms with standard deviation for random effects variance (REV). Hard coral cover is fixed effects and Year represents random effects. Significance codes: ‘***’ 0.001 ‘**’ 0.01 ‘*’ 0.05.
(DOCX)

S2 Table Summary of zero-inflated negative binomial generalized linear mixed effect model showing the effect of hard coral cover and year on fish abundance. The table shows the best-selected model indicating parameter means with standard errors for fixed effects (FEM), and variance terms with standard deviation for random effects (REV). Hard coral cover is fixed effects and Year represents random effects. Significance codes: ‘***’ 0.001 ‘**’ 0.01 ‘*’ 0.05.
(DOCX)
