# Supplementary material for: Citizen science in data and resource-limited areas: A tool to detect long-term ecosystem changes
Source: PLoS One. 2019 Jan 9;14(1):e0210007. doi: 10.1371/journal.pone.0210007 (PMC6326458; doi:10.1371/journal.pone.0210007)

S1 Fig. Yearly changes per transect in mean fish community abundance and mean cover of hard coral, algae, soft coral and abiotic benthos (vertical lines represent standard error). Occurrence of one Degree Heating Month (DHM) displayed for each corresponding year.


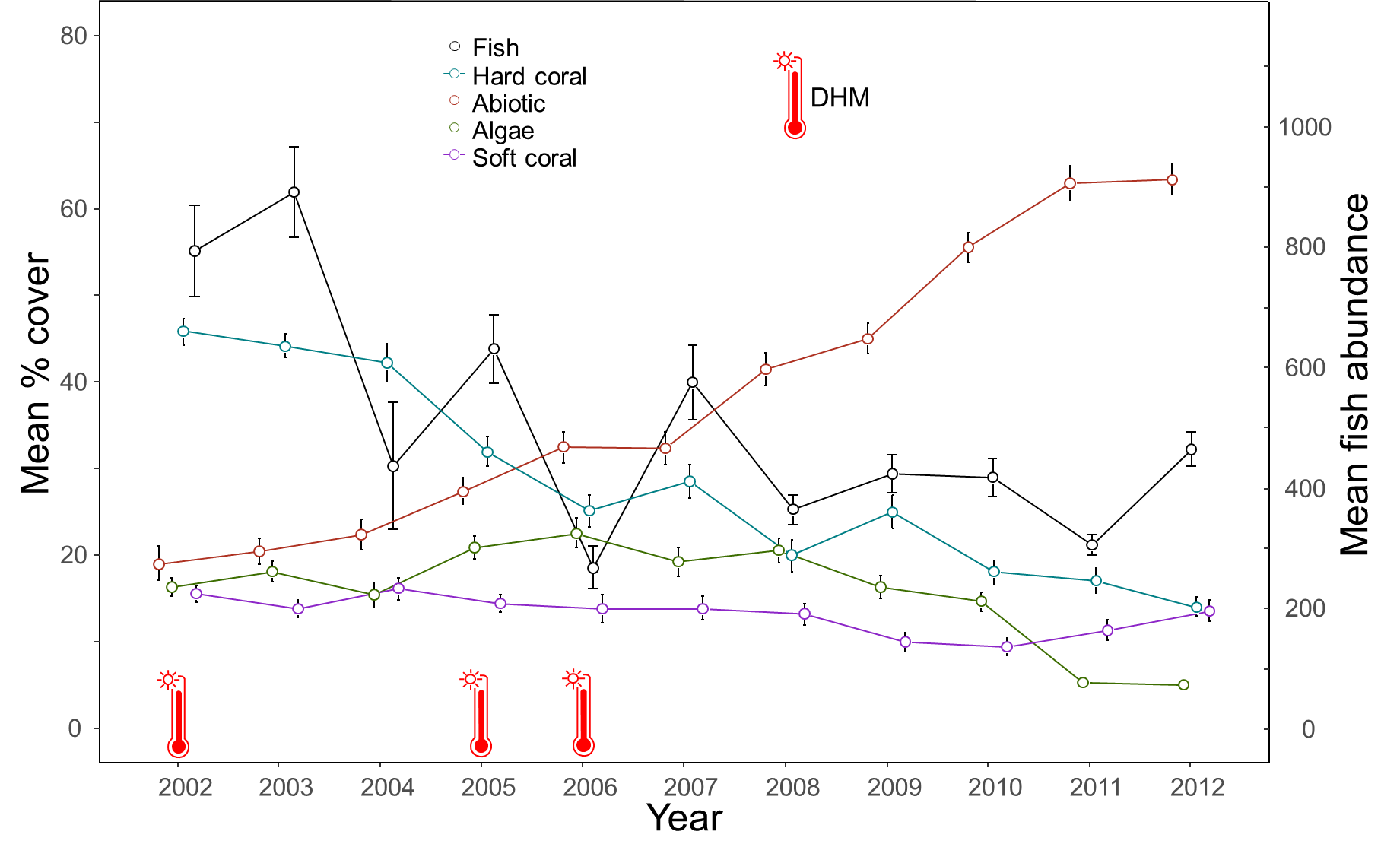

Supplement: S1 Fig — Occurrence of one Degree Heating Month (DHM) displayed for each corresponding year. (DOCX) [file pone.0210007.s001.docx]
