# Supplementary material for: Citizen science in data and resource-limited areas: A tool to detect long-term ecosystem changes
Source: PLoS One. 2019 Jan 9;14(1):e0210007. doi: 10.1371/journal.pone.0210007 (PMC6326458; doi:10.1371/journal.pone.0210007)
Supplement: S2 Fig — Negative binomial generalized linear mixed effect model fitted to the relationship between fish community a) abundance and b) species richness and hard coral cover for each year separately (solid grey lines) and mean of all years (solid black line) and confidence intervals of the mean (dashed black lines). Grey circles represent data points. (DOCX) [file pone.0210007.s002.docx]

S2 Fig. Negative binomial generalized linear mixed effect model fitted to the relationship between fish community a) abundance and b) species richness and hard coral cover for each year separately (solid grey lines) and mean of all years (solid black line) and confidence intervals of the mean (dashed black lines). Grey circles represent data points.
