# Supplementary material for: Citizen science in data and resource-limited areas: A tool to detect long-term ecosystem changes
Source: PLoS One. 2019 Jan 9;14(1):e0210007. doi: 10.1371/journal.pone.0210007 (PMC6326458; doi:10.1371/journal.pone.0210007)

**S4 Fig.** Zero-inflated negative binomial generalized linear mixed effect models fitted to the relationship between fish families’ abundance and hard coral cover for each year separately (solid grey lines) and mean of all years (solid black line) and confidence intervals of the mean (dashed black lines): A) Nemipteridae, B) Pomacanthidae, C) Pomacentridae, D) Pseudochromidae, E) Zanclidae.


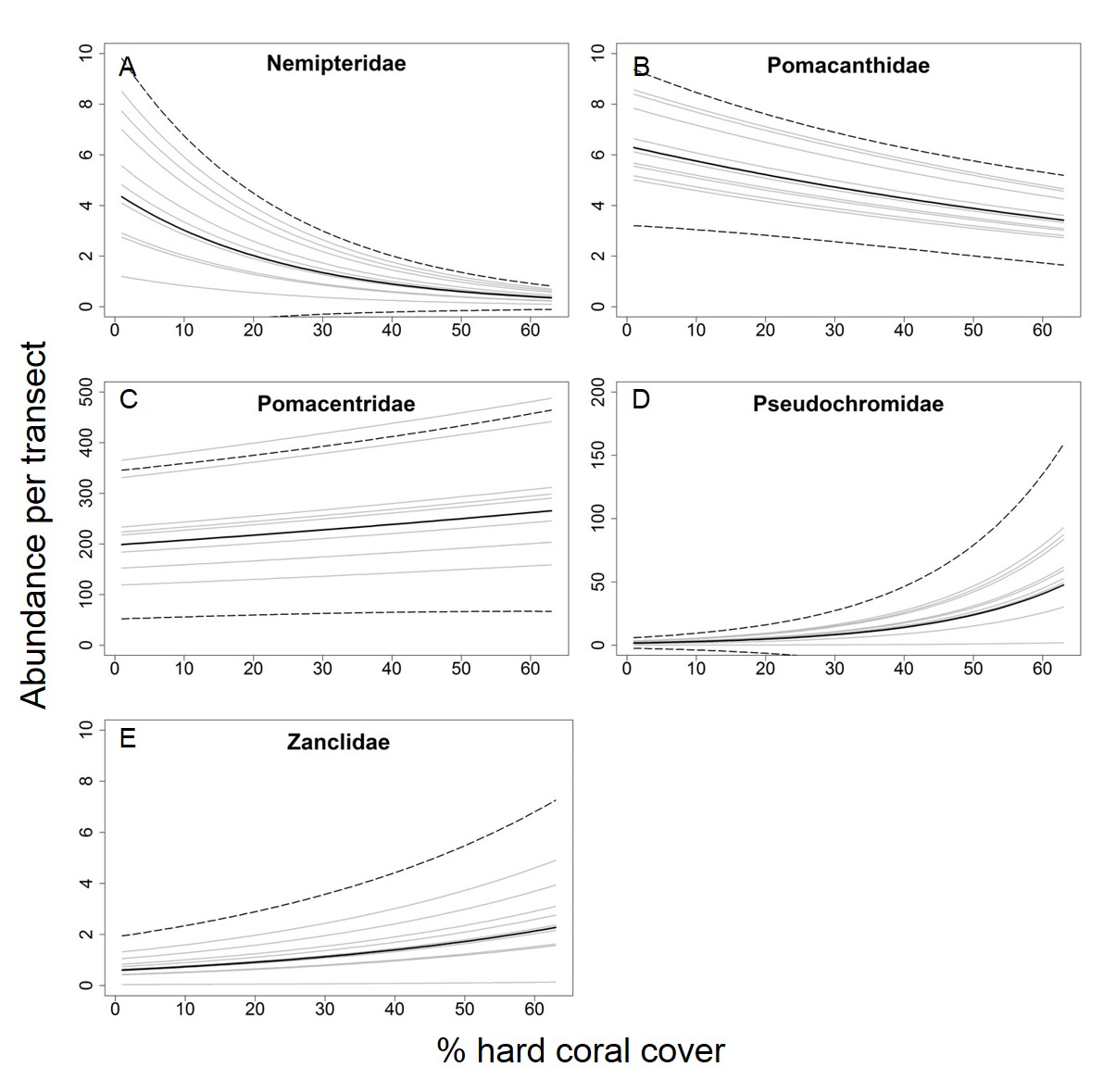

Supplement: S4 Fig — Zero-inflated negative binomial generalized linear mixed effect models fitted to the relationship between fish families’ abundance and hard coral cover for each year separately (solid grey lines) and mean of all years (solid black line) and confidence intervals of the mean (dashed black lines): A) Nemipteridae, B) Pomacanthidae, C) Pomacentridae, D) Pseudochromidae, E) Zanclidae. (DOCX) [file pone.0210007.s004.docx]
