# Supplementary material for: Citizen science in data and resource-limited areas: A tool to detect long-term ecosystem changes
Source: PLoS One. 2019 Jan 9;14(1):e0210007. doi: 10.1371/journal.pone.0210007 (PMC6326458; doi:10.1371/journal.pone.0210007)
Supplement: S5 Fig — Statistically significant zero-inflated negative binomial generalized linear mixed effect models for the relationship between species’ abundance and hard coral cover for each year separately (solid grey lines) and mean of all years (solid black line) with the mean confidence intervals (dashed black lines): A) Acanthuridae, B) Anthinae, C) Balistidae, D) Chaetodontidae, E) Labridae, F) Pomacanthidae, G) Pomacentridae and H) Pseudochromidae species. (DOCX) [file pone.0210007.s005.docx]

S5 Fig. Statistically significant zero-inflated negative binomial generalized linear mixed effect models for the relationship between species’ abundance and hard coral cover for each year separately (solid grey lines) and mean of all years (solid black line) with the mean confidence intervals (dashed black lines): A) Acanthuridae, B) Anthinae, C) Balistidae, D) Chaetodontidae, E) Labridae, F) Pomacanthidae, G) Pomacentridae and H) Pseudochromidae species.
