# Supplementary material for: Citizen science in data and resource-limited areas: A tool to detect long-term ecosystem changes
Source: PLoS One. 2019 Jan 9;14(1):e0210007. doi: 10.1371/journal.pone.0210007 (PMC6326458; doi:10.1371/journal.pone.0210007)
Supplement: S1 Table — The table shows the best-selected model indicating parameter means with standard errors for fixed effects mean (FEM), and variance terms with standard deviation for random effects variance (REV). Hard coral cover is fixed effects and Year represents random effects. Significance codes: ‘***’ 0.001 ‘**’ 0.01 ‘*’ 0.05. (DOCX) [file pone.0210007.s006.docx]

S1 Table. Summary of zero-inflated negative binomial generalized linear mixed effect model showing the effect of hard coral cover and year on fish family abundance. The table shows the best-selected model indicating parameter means with standard errors for fixed effects mean (FEM), and variance terms with standard deviation for random effects variance (REV). Hard coral cover is fixed effects and Year represents random effects. Significance codes: ‘***’ 0.001 ‘**’ 0.01 ‘*’ 0.05.

| Selected model: fish abundance ~ hard coral + random (year) | | | | | |
| --- | --- | --- | --- | --- | --- |
| FAMILY |  | FEM ± (SE) | z statistic | P | REV ± (SE) |
| ACANTHURIDAE | intercept | 3.73(0.17) | 22.46 | *** | 0.21(0.46) |
|  | hard coral | -0.01(0) | -3.24 | ** |  |
| ANTHINAE | intercept | 3.13(0.34) | 9.22 | *** | 0.2(0.45) |
|  | hard coral | 0.03(0.01) | 3.8 | *** |  |
| BALISTIDAE | intercept | 0.84(0.3) | 2.82 | ** | 0.52(0.72) |
|  | hard coral | 0.04(0.01) | 5.9 | *** |  |
| CHAETODONTIDAE | intercept | 2.35(0.11) | 21.7 | *** | 0.05(0.22) |
|  | hard coral | 0.02(0) | 5.57 | *** |  |
| HOLOCENTRIDAE | intercept | 0.01(0.23) | 0.03 | 0.06 | 0.06(0.25) |
|  | hard coral | -0.03 | 0.01 | *** |  |
| LABRIDAE | intercept | 3.07(0.16) | 19.28 | *** | 0.18(0.42) |
|  | hard coral | 0.01(0) | 2.04 | * |  |
| LUTJANIDAE | intercept | -0.25(0.29) | -0.85 | 0.27 | 0.27(0.52) |
|  | hard coral | 0.04(0.01) | 5.27 | *** |  |
| NEMIPTERIDAE | intercept | 1.51(0.26) | 5.92 | *** | 0.35(0.59) |
|  | hard coral | -0.04(0.01) | -6.29 | *** |  |
| POMACANTHIDAE | intercept | 1.85(0.12) | 14.96 | *** | 0.05(0.22) |
|  | hard coral | -0.01(0) | -2.8 | ** |  |
| POMACENTRIDAE | intercept | 5.33(0.13) | 39.91 | 0.35 | 0.12(0.35) |
|  | hard coral | 0(0) | 1.72 | * |  |
| PSEUDOCHROMIDAE | intercept | 0.58(0.42) | 1.37 | 1.11 | 1.22(1.11) |
|  | hard coral | 0.05(0.01) | 6.61 | *** |  |
| SERRANIDAE | intercept | 0.99(0.27) | 3.67 | *** | 0.51(0.71) |
|  | hard coral | -0.02(0.01) | -3.52 | *** |  |
| ZANCLIDAE | intercept | -0.18(0.38) | -0.47 | 0.11 | 1.11(1.05) |
|  | hard coral | 0.02(0.01) | 3.9 | *** |  |
